# Molybdenum-Induced Effects on Nitrogen Metabolism Enzymes and Elemental Profile of Winter Wheat (*Triticum aestivum* L.) Under Different Nitrogen Sources

**DOI:** 10.3390/ijms20123009

**Published:** 2019-06-20

**Authors:** Muhammad Imran, Xuecheng Sun, Saddam Hussain, Usman Ali, Muhammad Shoaib Rana, Fahd Rasul, Muhammad Hamzah Saleem, Mohamed G. Moussa, Parashuram Bhantana, Javaria Afzal, Ali Mohamed Elyamine, Cheng Xiao Hu

**Affiliations:** 1 Key Laboratory of Arable Land Conservation (Middle and Lower Reaches of Yangtze River), Ministry of Agriculture and Rural Affairs, Huazhong Agricultural University, Wuhan 430070, China; imrangorayauaf@yahoo.com (M.I.); sxccn@mail.hzau.edu.cn (X.S.); muhammadshoaib1555@gmail.com (M.S.R.); mohamedgomaa_ali@agr.asu.edu.eg (M.G.M.); pabh@webmail.hzau.edu.cn (P.B.); juvaria_afzal@outlook.com (J.A.); elyoh@hotmail.fr (A.M.E.); 2 Microelement Research Center, Huazhong Agricultural University, Wuhan 430070, China; 3 Department of Agronomy, University of Agriculture Faisalabad, 38040 Punjab, Pakistan; sadamhussainuaf@gmail.com (S.H.); drfahdrasul@gmail.com (F.R.); 4 College of Plant Science and Technology, Huazhong Agricultural University, Wuhan 430070, China; usali@student.qau.edu.pk (U.A.); saleemhamza312@webmail.hzau.edu.cn (M.H.S.); 5 Soil and Water Research Department, Nuclear Research Center, Egyptian Atomic Energy Authority, Abou Zaabl 13759, Egypt; 6 Faculty of Science and Technology, Department of Life Science, University of Comoros, Moroni 269, Comoros

**Keywords:** molybdenum, nitrogen sources, nitrogen metabolism enzymes, mineral elements, winter wheat

## Abstract

Different nitrogen (N) sources have been reported to significantly affect the activities and expressions of N metabolism enzymes and mineral elements concentrations in crop plants. However, molybdenum-induced effects in winter wheat cultivars have still not been investigated under different N sources. Here, a hydroponic study was carried out to investigate these effects on two winter wheat cultivars (‘97003’ and ‘97014’) as Mo-efficient and Mo-inefficient, respectively, under different N sources (NO_3_^−^, NH_4_NO_3_, and NH_4_^+^). The results revealed that the activities of nitrate reductase (NR) and nitrite reductase (NiR) followed the order of NH_4_NO_3_ > NO_3_^−^ > NH_4_^+^ sources, while glutamine synthetase (GS) and glutamate synthase (GOGAT) followed the order of NH_4_^+^ > NH_4_NO_3_ > NO_3_^−^ in both the wheat cultivars. However, Mo-induced effects in the activities and expressions of N metabolism enzymes under different N sources followed the order of NH_4_NO_3_ > NO_3_^−^ > NH_4_^+^ sources, indicating that Mo has more complementary effects towards nitrate nutrition than the sole ammonium source in winter wheat. Interestingly, under −Mo-deprived conditions, cultivar ‘97003’ recorded more pronounced alterations in Mo-dependent parameters than ‘97014’ cultivar. Moreover, Mo application increased the proteins, amino acids, ammonium, and nitrite contents while concomitantly decreasing the nitrate contents in the same order of NH_4_NO_3_ > NO_3_^−^ > NH_4_^+^ sources that coincides with the Mo-induced N enzymes activities and expressions. The findings of the present study indicated that Mo plays a key role in regulating the N metabolism enzymes and assimilatory products under all the three N sources; however, the extent of complementation exists in the order of NH_4_NO_3_ > NO_3_^−^ > NH_4_^+^ sources in winter wheat. In addition, it was revealed that mineral elements profiles were mainly affected by different N sources, while Mo application generally had no significant effects on the mineral elements contents in the winter wheat leaves under different N sources.

## 1. Introduction

Plants take nitrogen (N) either as nitrate (NO_3_^−^) or ammonium (NH_4_^+^) form for various growth and developmental processes; however, NO_3_^−^ is more important for such processes. For most of the crop plants, the NO_3_^−^ form is mobile, less toxic, and can be stored in vacuoles. However, NO_3_^−^ must be reduced to NH_4_^+^ before it can be utilized for the synthesis of amino acids, proteins, and other nitrogenous compounds in plant cells. Nitrate reductase (NR) and nitrite reductase (NiR), the key nitrate assimilatory enzymes, are located in cytosol and chloroplasts and catalyze NO_3_^−^ reduction to NO_2_^−^ followed by NO_2_^−^ to NH_4_^+^, respectively, in the leaf tissues [1]. However, NH_4_^+^ is directly assimilated to produce different amino acids by the mutual actions of glutamine synthetase (GS) and glutamate synthase (GOGAT) enzymes in a cyclic manner within the plant cells [2].

In plants, NO_3_^−^ is reduced to NO_2_^−^ by the activities of the NR enzyme present in the cytosols of cells. However, NR activities are largely dependent on the molybdenum cofactor (Moco), nitrate ions, hormones, growth conditions, reduced N metabolites, and phosphorylation [3,4,5]. Plant cells can store NO_3_^−^ without toxic effects, but on the contrary, NO_2_^−^ is highly toxic and needs immediate metabolization. NiR is responsible for catalyzing the six-electron reduction of NO_2_^−^ to NH_4_^+^. However, the conversion rate of NO_2_^−^ to NH_4_^+^ and NiR activity depend upon the repressive effects of reduced N metabolites such as NH_4_^+^ and amino acids contents [6].

Relative to the NO_3_^−^ form of N, NH_4_^+^ is highly toxic and needs instant detoxification to various organic nitrogenous compounds. The main mechanism of NH_4_^+^ detoxification in higher plants is the assimilation of NH_4_^+^ to different amino acids and amides through the combined activities of GS and GOGAT enzymes [7]. The GS has greater affinity towards NH_4_^+^ and therefore, even at lower concentrations, can assimilate NH_4_^+^ to avoid its buildup to phytotoxic levels. In the NH_4_^+^ assimilating process, GS plays a key role in catalyzing the glutamate *γ*-carboxyl group fixation to glutamine [8,9,10]. GOGAT is also involved in the NH_4_^+^ assimilation [11,12] and catalyzes the process of glutamic acid formation in which NH_4_^+^ ions enter into the nitrogenous compounds. Accordingly, these aminotransferases transfer the NH_4_^+^ assimilated glutamates to suitable *α*-ketoacids to form *α*-amino acids. These amino acids are then further assimilated to different nitrogenous compounds such as nucleic acids and proteins [13,14].

Molybdenum (Mo) is an essential microelement for higher plants and also a metal component of the Mo-cofactor, (Moco) biosynthesis. Moco binds to Mo-requiring enzymes and optimizes their activities for normal functioning of plant growth and developmental processes. Mo plays a significant role in N metabolism, which includes nitrate reduction, assimilation, and fixation, by regulating the NR and GS enzymes activities and expressions [15,16,17]. Moreover, previous studies have reported that Mo-deficiencies are predominantly associated with poor N health and the plants show symptoms similar to N deficiencies [18], indicating that Mo has a key role in the N metabolism.

The proper functioning of every living cell is only possible through the availability of essential macro- and micro-elements. A critical network of gene products controls complex processes for the uptake, binding, transportation, and repossession of a given element in the plant cells [19]. Ionomics revealed that changes in the macro- or micro-element nutritional status of plants are associated with changes in a given subset of elements [20,21]. Different N sources available in the rhizosphere considerably affected the mineral element profiles of both macro and micro-elements in leaves and consequently affected the N metabolism, photosynthetic rate, growth, and yields in *Citrus sinensis* [22], cucumber [23], and watermelon [24].

Wheat (*Triticum aestivum* L.), the second most widely grown crop over the world, has a specific preference for NO_3_^−^ source and shows toxicity symptoms under NH_4_^+^ source. However, nitrate buildups in wheat grains have serious consequences for human health because excess NO_3_^−^ consumption can increase the risk of cancer in adults, and serious health damage, especially in children. It can cause methaemoglobinaemia, a type of rare but potentially fatal haemoglobinopath [25]. In nitrate-induced methaemoglobinaemia, dietary nitrate is reduced to nitrite in the stomach, and the absorbed nitrite then converts hemoglobin to methemoglobin in red blood cells by oxidizing the heme Fe_2_^+^ ion to Fe_3_^+^ [26]. This oxidation prevents methemoglobin from binding oxygen and compromises oxygen delivery to peripheral tissues. So, methaemoglobinaemia underlines the importance of optimal N metabolism in leaf tissues, which are actually the grain formation sources in crop plants, especially in wheat, which is the staple food in most countries, and whose optimal N metabolism could be achieved by regulating N metabolism enzymes activities and expressions.

Most of the previous and recent studies have repeatedly focused and reported the Mo and NO_3_^−^ interactions in different crop plants [16,27,28,29,30,31]; however, the effects of Mo application on the N metabolism of winter wheat leaves have still not been reported under different N sources. Therefore, in the current study we investigated Mo-induced effects on N metabolism enzymes activities, gene expression patterns, N assimilatory products, and macro- and micro-elements contents in winter wheat leaves under different N sources and also highlighted the extent of complementation between these fertilizers.

## 2. Results

### 2.1. Effects of Mo Application on N Metabolism Enzymes Activities Under Different N Sources

**Figure 1:**
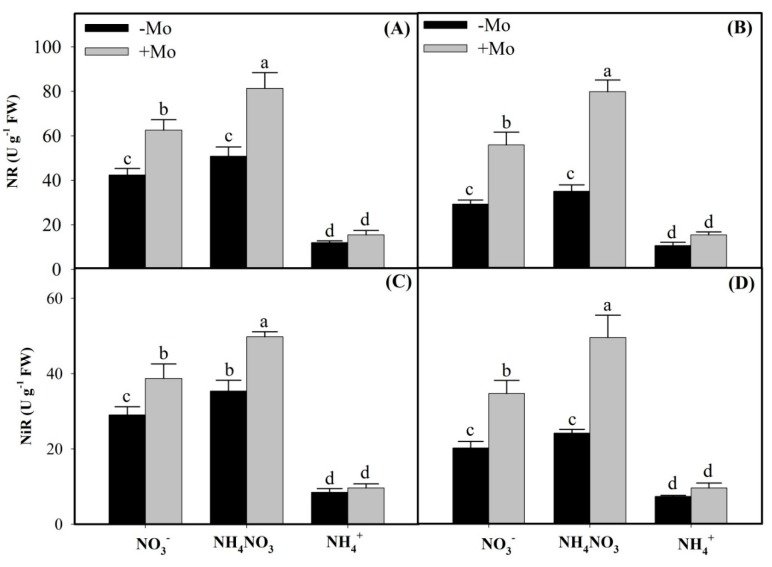
Impacts of Molybdenum (Mo) application on nitrate reductase (NR) and nitrite reductase (NiR) activities of Mo-efficient winter wheat ‘97003’ cultivar (**A**,**C**) and Mo-inefficient winter wheat ‘97014’ cultivar (**B**,**D**) under different N sources. −Mo and +Mo treatments represent the 0 and 1 µM Mo [Na_2_MoO_4_.2H_2_O] concentrations, respectively, in Hoagland solution. Vertical bar above indicates standard error of four replicates. Different lowercase letters (a, b, c, etc.) represent significant differences according to the least significant difference (LSD)-test (*p* < 0.05, *n* = 4). NO_3_^−^: sole nitrate source, NH_4_NO_3_: co-applied ammonium nitrate, NH_4_^+^: sole ammonium source.

**Figure 2:**
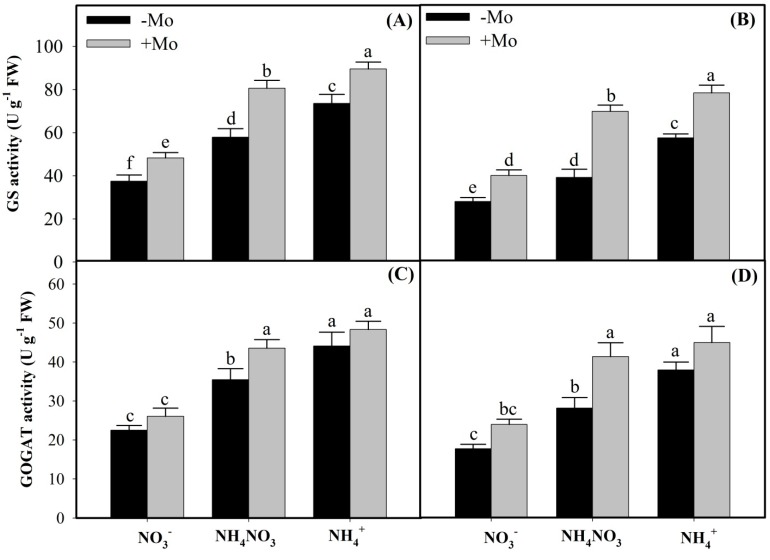
Impacts of Molybdenum (Mo) application on glutamine synthetase (GS) and glutamate synthase (GOGAT) activities in the leaves of Mo-efficient winter wheat ‘97003’ cultivar (**A**,**C**) and Mo-inefficient ‘97014’ cultivar (**B**,**D**) under different N sources. −Mo and +Mo treatments represent the 0 and 1 µM Mo [Na_2_MoO_4_.2H_2_O] concentrations, respectively, in Hoagland solution. Vertical bar above indicates standard error of four replicates. Different lowercase letters (a, b, c, etc.) represent significant differences according to the LSD-test (*p* < 0.05, *n* = 4). Description of treatments is mentioned in Figure 1.

In the present study, under different N sources, NR and NiR enzymes activities followed the order of NH_4_NO_3_ > NO_3_^−^ > NH_4_^+^ sources, whereas GS and GOGAT enzymes activities followed the order of NH_4_^+^ > NH_4_NO_3_ > NO_3_^−^ in both the winter wheat cultivars (Figure 1 and Figure 2). However, Mo application resulted in considerable increases of NR and NiR activities in NO_3_^−^and NH_4_NO_3_ sources, except that non-significant effects were observed under sole NH_4_^+^ environment in both the winter wheat cultivars (Figure 1). Interestingly, compared with −Mo plants, Mo application significantly enhanced the GS activities under all N sources in both the wheat cultivars, with Mo-inefficient ‘97014’ cultivar showing more pronounced increases than the Mo-efficient ‘97003’ cultivar (Figure 2A,B), suggesting that Mo-inefficient ‘97014’ cultivar is comparatively more dependent on the external Mo supply than the Mo-efficient ‘97003’ cultivar when regulating the N metabolism enzymes. However, Mo supply did not significantly increase the GOGAT activities under sole either NO_3_^−^-N or NH_4_^+^-N sources, except for the NH_4_NO_3_ source in both the wheat cultivars (Figure 2C,D), suggesting that Mo-induced effects in regulating the N metabolism of winter wheat plants under different N sources are more harmonious when available in mixture supply as NH_4_NO_3_ than as sole applications of either N source in wheat plants.

### 2.2. Mo Supply Regulated the Expressions of NR, NiR, GS, and GOGAT Genes

**Figure 3:**
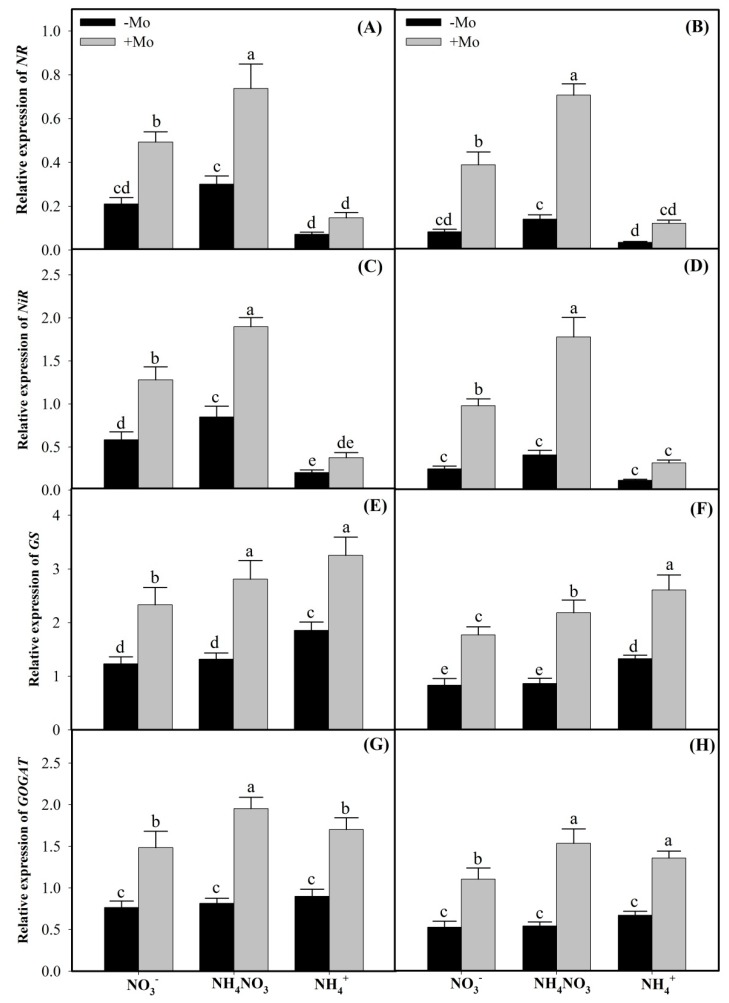
qRT-PCR analysis of *NR, NiR, GS,* and *GOGAT* genes transcripts in the leaves of Mo-efficient ‘97003’ (**A**,**C**,**E**,**G**) and Mo-inefficient ‘97014’ (**B**,**D**,**F**,**H**) winter wheat cultivars. Seedlings were grown in nutrient solutions supplied with or without Mo fertilizer [Na2MoO4.2H2O] under different N sources. −Mo and +Mo treatments represent the 0 and 1 µM Mo concentrations, respectively, in Hoagland solution. Vertical bar above indicates standard error of four replicates. Different lowercase letters (a, b, c, etc.) represent significant differences according to the LSD-test (*p* < 0.05, *n* = 4). Description of treatments is mentioned in Figure 1.

Among different N sources, the transcript abundance of *NR* and *NiR* genes followed the order of NH_4_NO_3_ > NO_3_^−^-N > NH_4_^+^-N sources in both the winter wheat cultivars (Figure 3A–D). Compared with the −Mo-treated plants, +Mo application significantly up-regulated the expressions of *NR* and *NiR* genes under NO_3_^−^-N and NH_4_NO_3_ sources relative to sole NH_4_^+^-N source in both the winter wheat cultivars (Figure 3A–D). However, in contrast to *NR* and *NiR* genes, Mo application also significantly up-regulated the expressions of *GS* and *GOGAT* genes under sole NH_4_^+^ source (Figure 3E–H). Interestingly, Mo-efficient winter wheat ‘97003’ cultivar recorded more pronounced Mo-dependent expressions than Mo-inefficient ‘97014’ cultivar under Mo-deprived environment (Figure 3), indicating that ‘97003’ cultivar can proficiently withstand Mo-deficient conditions than ‘97014’ winter wheat cultivar.

### 2.3. Effects of Mo Application on Inorganic and Reduced N Accumulation

In the present study, compared with −Mo plants, Mo application significantly decreased the NO_3_^−^ contents in ‘97014’ cultivar relative to ‘97003’ cultivar under NO_3_^−^ and NH_4_NO_3_ sources (Table 1). However, in contrast to NO_3_^−^ contents, Mo application increased the NO_2_^−^ and NH_4_^+^ contents in both the winter wheat cultivars (Table 1), suggesting that Mo plays a significant role in efficient NO_3_^−^ assimilation pathways.

**Figure 4:**
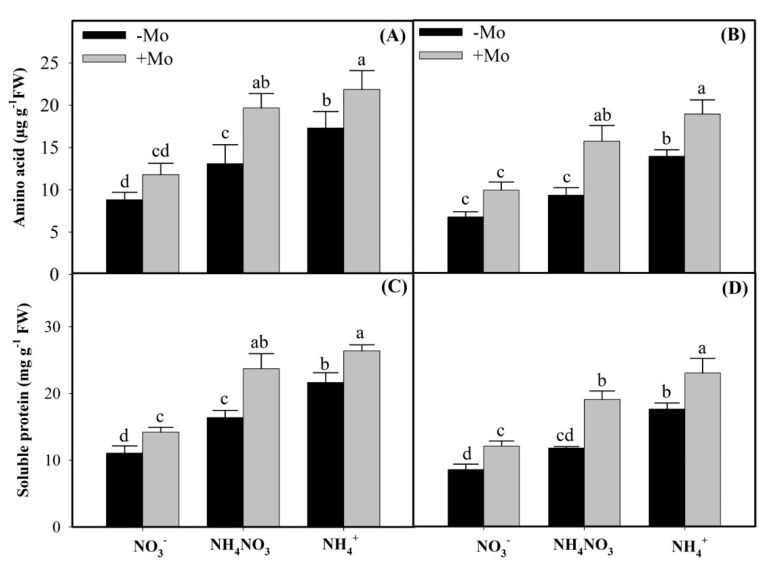
Effects of Molybdenum (Mo) application on amino acids and soluble protein contents in leaves of Mo-efficient winter wheat ‘97003’ cultivar (**A**,**C**) and Mo-inefficient winter wheat ‘97014’ cultivar (**B**,**D**) under different N sources. −Mo and +Mo treatments represent 0 and 1 µM Mo [Na_2_MoO_4_.2H_2_O] concentrations, respectively, in Hoagland solution. Vertical bar above indicates standard error of four replicates. Different lowercase letters (a, b, c, etc.) represent significant differences according to the LSD-test (*p* < 0.05, *n* = 4). Description of treatments is mentioned in Figure 1.

Amino acids and soluble proteins contents under different N sources followed the order of NH_4_^+^ > NH_4_NO_3_ > NO_3_^−^ in both the winter wheat cultivars (Figure 4). Compared with the −Mo treatments, Mo application increased the amino acids and soluble proteins contents in all N sources; however, Mo-induced increases under different N sources were highest under NH_4_NO_3_ source than sole application of either N source (Figure 4), suggesting that Mo is harmonious with N when applied in mixture form as NH_4_NO_3_ source in winter wheat. Moreover, under Mo deficient conditions, Mo-efficient ‘97003’ cultivar showed more pronounced increases than Mo-inefficient ‘97014’ winter wheat cultivar (Figure 4), validating that Mo-efficient winter wheat ‘97003’ cultivar could comparatively withstand Mo-deficient soil conditions with less harm to Mo-dependent processes.

### 2.4. Effects of Mo Application on the Elemental Profiling of Winter Wheat Leaves Under Different N Sources

The effects of Mo supply on the macro-elements (K, Mg, and Ca) and micro-elements (Mn, Zn, Cu, and Fe) concentrations in the leaf tissues of winter wheat cultivars under various N forms are given in Table 2 and Table 3, respectively. In the present study, the concentrations of K, Ca, Mg, and Mn in the leaf tissues of both the winter wheat cultivars under different N sources followed the order of NO_3_^−^ > NH_4_NO_3_ > NH_4_^+^ sources (Table 2 and Table 3). However, compared with the −Mo plants, the Ca and Mg concentrations were higher in the +Mo-treated plants under all N sources, while in contrast K and Mn concentrations were reduced in the wheat leaves (Table 2 and Table 3). Moreover, in the present study, Zn, Cu, and Fe concentrations under different N sources followed the order of NH_4_^+^ > NH_4_NO_3_ > NO_3_^−^ in both the wheat cultivars (Table 3). However, compared with the −Mo treatments, Mo supply decreased the Mn, Zn, and Fe concentrations in the leaf tissues, except for Cu in both the winter wheat leaves (Table 3).

## 3. Discussion

Nitrate and ammonium are the major forms of N that plants use for different growth and developmental processes. The form of N available in the rhizosphere significantly affects N reduction, assimilation, gene expression patterns, N metabolism enzymes activities and assimilatory products, and the elemental profile of macro- and micro-elements [23,24,32,33,34]. Despite the fact that more energy is needed for NO_3_^−^ assimilation, most of the crop plants prefer NO_3_^−^ over NH_4_^+^ as their primary N source [35,36]. NO_3_^−^ form of N is first converted to NO_2_^−^ and then to NH_4_^+^ by the sequential actions of NR and NiR enzymes, whereas NH_4_^+^ is directly assimilated into amino acids via the concerted activities of GS and GOGAT enzymes [37]. So, the present study investigated, for the first time, the influence of Mo application on the activities and expressions of NR, NiR, GS, and GOGAT enzymes; assimilatory products; and the elemental profile in winter wheat leaves under different N sources.

In the present study, NO_3_^−^ and NH_4_NO_3_ sources increased the NO_3_^−^ contents in the wheat leaves (Table 1). In plant root systems, constitutive low-affinity transporter and inducible high-affinity transport systems are reserved to absorb NO_3_^−^ from soil solutions. However, regulation of these transport systems is dependent on intracellular NO_3_^−^ consumptions and cellular energy supplies [6,38]. Accumulated NO_3_^−^ is reduced to NO_2_^−^ through NR activities. In our study, Mo application significantly increased NR activities and expressions under NO_3_^−^-N and NH_4_NO_3_ sources, compared to sole NH_4_^+^-N source (Figure 1A,B), which might be due to the fact that induction of NR in plants requires both NO_3_^−^ and Mo elements; if either nutrient is deficient, the enzyme is either non-existent or less active [39]. Furthermore, we observed decreased NO_3_^−^ and increased NO_2_^−^ and NH_4_^+^ contents in the leaf tissues of both the winter wheat cultivars, with Mo-inefficient ‘97014’ cultivar showing more pronounced effects than the Mo-efficient ‘97003’ cultivar upon Mo application (Table 1). However, these differential responses between the two cultivars are consistent with the previous reports where Mo-efficient ‘97003’ cultivar yielded more than 90% and the Mo-inefficient ‘97014’ cultivar less than 50% under Mo deprivations when compared with the Mo applied (+Mo) treatments [40,41]. Nitrite contents depend upon NO_3_^−^ availability in the rhizosphere, and NR activities and their mRNA expressions [32]. Our findings indicated that NO_2_^−^ contents under different N sources followed the order of NH_4_NO_3_ > NO_3_^−^ > NH_4_^+^ sources. These findings might be due to the reason that Mo-induced NR activities and expressions under different N sources followed the same order as NH_4_NO_3_ > NO_3_^−^ > NH_4_^+^ in both the winter wheat cultivars. NR is a rate-limiting enzyme in the NO_3_^−^ reduction to NO_2_^−^ in higher plants [42], because it acts as a catalyst in the first step of the NO_3_^−^ reduction pathway, yielding NO_2_^−^, which in turn is further reduced to NH_4_^+^ [39,43]. Our findings that the increased NO_2_^−^ contents under Mo application coincide with the higher NR activities and expressions in the wheat leaves (Figure 1 and Figure 3A,B) are in accordance with previous studies in soybean [44], common bean [45], maize [31], and strawberry [16].

Plants accumulate NH_4_^+^ either directly through ammonium transporters and/or they produce it in the NO_3_^−^ reduction pathways [46,47,48]. In the present study, in contrast to NO_3_^−^ contents, Mo supply increased the NO_2_^−^ and NH_4_^+^ contents under all N sources in the leaves of wheat plants (Table 1). Therefore, these observations suggest that higher NH_4_^+^ contents, under sole NH_4_^+^ source, might be due to the ammonium transporters because these are highly responsive to NH_4_^+^ availability [42,49] while in NO_3_^−^ and NH_4_NO_3_ sources through Mo-induced higher activities and expressions of NR and NiR enzymes (Figure 1 and Figure 3). These results agree with the previous reports that reduced NR activities and their mRNA expressions reduce the NO_2_^−^ contents in leaf tissues [16,50,51]. Similarly, Mo-induced NiR activity and its mRNA expressions and concomitantly higher NH_4_^+^ contents in this study coincide with a direct relationship that exists between NiR activity and its transcript abundance, and the NH_4_^+^ accumulations [32].

GS and GOGAT play a significant role in the direct assimilation of NH_4_^+^ to amino acids in the leaves of higher plants [32,37]. Results of the present study showed that GS and GOGAT activities and expressions, and concomitantly amino acids and soluble protein contents under different N sources, followed the order of NH_4_^+^ > NH_4_NO_3_ > NO_3_^−^ in both the winter wheat leaves. The reason might be that lower concentrations of external NO_3_^−^ increase the GS and GOGAT activities [9], while higher cytoplasmic NO_3_^−^ contents diminish their activities, along with reducing the amino acids and soluble protein contents [9,52]. However, Mo-induced increases in the GS and GOGAT activities and expressions, and similarly amino acids and soluble proteins contents under different N sources, followed the order of NH_4_NO_3_ > NO_3_^−^ > NH_4_^+^ sources (Figure 2 and Figure 3E–F), suggesting that Mo has more complementary effects towards N when applied in the mixture form as NH_4_NO_3_ than the sole application of either source in winter wheat. Similar results have also been reported previously (that Mo application significantly enhanced the GS and GOGAT activities and expressions [15,16] and free amino acids and soluble protein contents in winter wheat leaves [53,54]).

We also observed that the mineral elements concentrations in the leaf tissues were markedly influenced by various N forms. The concentrations of macro-elements K, Ca, and Mg under different N sources followed the order of NO_3_^−^ > NH_4_NO_3_ > NH_4_^+^ in both the winter wheat cultivars (Table 2). The reason is that translocation rates of K, Mg, and Ca might have reduced in the xylem sap of the wheat plants supplied with sole NH_4_^+^ source due to lower demand of cations for charge balance as previously observed [55]. Similar observations have also been reported in maize [55], cucumber [23], *Arabidopsis thaliana* [56], and watermelon [24]. However, microelements concentrations (Zn, Cu, and Fe except Mn), under different N sources followed the order of NH_4_^+^ > NH_4_NO_3_ > NO_3_^−^ sources in both the winter wheat leaves (Table 3). These exceptional observations for Mn accumulation, compared to the other micronutrients, have also been reported previously in citrus plants [22]. The reason might be that re-translocation, recirculation, tissue internal demand, environmental conditions, and different plant species are the factors affecting the minerals uptake in plants [55,57]. Generally, compared with −Mo plants, Mo supply did not significantly affect the macro- or micro-elements concentrations in the winter wheat leaves under different N sources. Similar results have been reported in the root and leaf ionomes of cucumber plants grown with or without Mo fertilizer under hydroponic environment [58].

Taken together, the results of the present study conclude that Mo played a key role in improving the N metabolism under all the three N sources. However, Mo-induced effects on the N metabolism enzymes and assimilatory products exist in the order of NH_4_NO_3_ > NO_3_^−^ > NH_4_^+^, suggesting that Mo is more harmonious with N when available in the mixture supply as NH_4_NO_3_ source than sole application of either source in winter wheat. However, more pronounced effects, in the Mo-dependent parameters, observed in the Mo-efficient winter wheat cultivar under −Mo conditions suggest that this cultivar might better adapt to Mo-deficient conditions with less harm to the N metabolism than the Mo-inefficient winter wheat cultivar.

## 4. Materials and Methods

### 4.1. Plant Materials and Growth Conditions

In the present experiment, two winter wheat cultivars, ‘97003’ (Mo-efficient) and ‘97014’ (Mo-inefficient), which are different in Mo uptake, distribution, and utilization efficiency [40,59,60], were obtained from the Laboratory of Trace Elements, Huazhong Agricultural University, China. Seeds were surface sterilized with a 0.5% sodium hypochlorite (NaOCl) solution and rinsed three times with sterile distilled water. The seeds were allowed to germinate in deionized water at 25 °C for five days. The conditions in the controlled environment chamber were 24/18 °C, 14/10 h day/night, 400 µmol·m^−2^·s^−1^ irradiance, and 60–70% relative humidity [53]. Uniform-sized wheat seedlings were transferred to plastic containers holding one-quarter strength Hoagland solution. Wheat seedlings were fixed into the perforated lids of the containers through small sponges. The wheat seedlings were grown in one-quarter and one-half strength Hoagland solutions for the first and second 2-day period, respectively, and then the seedlings were continuously supplied with a full-strength Hoagland solution for 30-days. A nitrification inhibitor (dicyandiamide, 8.0 µM) was added along with Hoagland solution to prevent NH_4_^+^ oxidation. The concentrations of three different N sources and macro-nutrients are mentioned in Table 4, and rests were added as follows: 100 µM EDTA-Fe, 0.8 µM ZnSO_4_·7H_2_O, 9.1 µM MnCl_2_·4H_2_O, 0.3 µM CuSO_4_·5H_2_O, and 46.2 µM H_3_BO_3_. Na_2_MoO_4_·2H_2_O was used as Mo fertilizer. Two Mo treatments, 0 (−Mo) and 1 µM (+Mo), were separately added with three N sources (Table 4).

### 4.2. Analysis of Nitrate Reductase Activity

The nitrate reductase (NR, EC 1.6.6.1) activity assay followed the protocol of our previous study [61]. Frozen plant leaf samples were ground in 4 mL cold 25 mM sodium phosphate (pH 8.7) buffer containing 1.3 mM EDTA and 10 mM cysteine and centrifuged at 4000 rpm and 4 °C for 15 min. The reaction mixture comprised of supernatant, 0.1 M KNO_3_, and 2.82 mM NADH. The reaction started with the addition of NADH and was then incubated for 30 min. The reaction was ended with 1% sulfanilamide and 0.02% N-phenyl-2-naphthylamine and left for 15 min. After centrifugation at 4000 rpm for 5 min, absorbance was determined at 540 nm.

### 4.3. Determination of Nitrite Reductase Activity

The nitrite reductase (NiR, EC 1.7.2.1) activity in the fresh leaves was determined according to the method of [62]. Briefly, the frozen leaf tissues were homogenized with cold 0.1 M potassium phosphate buffer (pH 7.5) and the reaction mixture was comprised of enzyme extract, 10 mM KNO_2_, 1.5% methylviologen, and 5% sodium dithionite (Na_2_S_2_O_4_) dissolved in 100 mM NaHCO_3_, whose addition initiated the reaction. The reaction mixture was nurtured at room temperature for 30 min and ended by the de-coloration of methylviologen. The nitrite contents were determined by measuring the absorbance at 540 nm in the solution consisting of supernatant, distilled water, 1% (*w*/*v*) N (1-naphty1)-ethylenediamine dihydrochloride, and 10% (*w*/*v*) sulfanilamide prepared in HCl.

### 4.4. Analysis of GS and NADH-GOGAT Activity

For the preparation of crude extracts, frozen leaves were homogenized with a pre-cooled extraction buffer consisting of 100 mM Tris–HCl (pH 7.6), 1.0 mM MgCl_2_-6H_2_O, 10 mM 2-mercaptoethanol, and 1.0 mM ethylenediaminetetraacetic acid (EDTA) in a mortar and pestle. The homogenates were centrifuged at 13,000 rpm for 15 min at 4 °C. The supernatants were collected as crude extracts for the measurement of GS and GOGAT enzymes assays in leaf tissues. The GS (EC 6.3.1.2) activity was measured in the leaf samples by following the method described by [63]. The crude enzyme extract was treated with synthase reaction fluid consisting of 0.1 M imidazole, 0.08 M MgSO_4_·7H_2_O, 0.02 M glutamic acid-Na, and 58 M hydroxylamine hydrochloride (pH 7.0) and 10 mM ATP and incubated for 15 min at 37 °C. The FeCl_3_ solution, consisting of 2% Tri-chloroacetic acid (TCA), 3.5% FeCl_3_, and 2% HCl, was added to terminate the reaction. The absorbance was then determined at 540 nm in spectrophotometer. The NADH-glutamate synthase (NADH-GOGAT; EC 1.4.1.14) activity was determined according to the method described by [64]. The crude enzyme extract was treated with 25 mM Tris-base, 100 mM α-Ketoglutaric acid, 10 mM KCl, 20 mM L-glutamine, and 3 mM NADH. Then, the absorbance was measured at 340 nm due to NADH oxidation.

### 4.5. Total RNA Extraction and Quantitative RT-PCR

The transcript abundance was measured in the frozen wheat leaves according to the method described by [61]. The total RNA was isolated and quantified with Nano Drop 2000 UV-VIS spectrophotometer (Thermo, Fisher, Waltham, MA, USA). The quantified RNA was then subjected to cDNA synthesis through reverse transcriptase using Oligo (dT18) primers, M-MLVRTase, and dNTP. A detection system of IQ5 Real-Time PCR (Bio-Rad, Hercules, CA, USA) was used to produce cDNA. The cDNA templates, the SYBR Green mix (Bio-Rad, United States), and gene-specific primers were mixed in a 96-well plate for the subsequent detections. The primers of *NR, NiR, GS,* and *GOGAT* genes were obtained from [32]. The wheat *Actin* gene was used as a reference for the genes of interest. The detailed information of primers is available in Table 5. The relative expression levels of the transcripts were calculated according to the method of [65].

### 4.6. Determination of Nitrate, Nitrite, and Ammonium Contents

Fresh wheat leaves were homogenized (1:10, *w*/*v*) in redistilled water, boiled for 15 min, and then filtered. Nitrate contents were determined according to the method of [66]. The reaction mixture was comprised of filtrate and 5% salicylic acid in concentrated H_2_SO_4_. After incubation for 15 min, 4M NaOH was added and absorbance was measured at 410 nm. A calibration curve was prepared for KNO_3_ and expressed in µmol NO_3_^−^·g^−1^·FW.

Nitrite contents were measured according the method described by [32]. Briefly, fresh wheat leaves were grinded with 10 mM Tris buffer (pH 7.4) containing 10% (*v*/*v*) glycerol, 2 mM EDTA, 2% (*w*/*v*) PVPP, and 1 mM DTT at 4 °C. The homogenate was centrifuged at 12,000× *g* for 15 min. Then, nitrite contents were analyzed by Griess reagent consisting of 0.1% naphthylethylene diamine dihydrochloride in H_2_O and 1% sulfanilamide in 2.5% H_3_PO_4_. Sodium nitrite was used as standard, and nitrite contents were expressed as μmol NO_2_^−^·g^−1^·FW.

Ammonium contents were measured using the Nessler reagent [67]. The reaction mixture was comprised of 0.1 mL filtrate, 0.01 mL 10% K–Na tartrate, 2.4 mL redistilled water, and 0.1 mL Nessler reagent. Spectrophotometer was used to measure the absorbance at 425 nm after 5 min. A standard calibration curve was prepared for NH_4_Cl and expressed in µmol NH_4_^+^ g^−1^·FW.

### 4.7. Measurement of Amino Acids

Free amino acid contents were measured according to method describe by [68]. Briefly, frozen wheat leaves were ground with phosphate buffer (pH 7.0) in a mortar and pestle. The reaction mixture was comprised of 10% pyridine and 2% ninhydrin solution. The absorbance was measured at 570 nm using spectrophotometer.

### 4.8. Measurement of Soluble Proteins

The total soluble protein contents in the leaf tissues were measured by following the method of [69] using bovine serum albumin (BSA). The absorbance was measured with spectrophotometer at 595 nm, and the pure reagent was used as blank.

### 4.9. Mineral Elements Assays

The plant samples were dried, grinded, weighed, and soaked with sulfuric acid and digested in a fume hood at 180 °C for 3 h, followed by the addition of H_2_O_2_. K concentration was then determined with a flame photometer (Model 410, Sherwood, USA).

For the determination of calcium (Ca); magnesium (Mg); and other micronutrients zinc (Zn), iron (Fe), manganese (Mn), and copper (Cu), dried plant samples were digested in 4:1 (*v*/*v*) HNO_3_:HClO_4_ (*v*/*v*) in a microwave oven (MLS 1200, Milestone, FKV, Italy). The elements (Ca, Mg, Zn, Fe, Mn, and Cu) concentrations in the digested samples were determined by Inductively Coupled Plasma-Mass Spectrometry (ICP-MS).

### 4.10. Statistical Analysis

Data were statistically analyzed following two-way analysis of variance (ANOVA) using Statistix 8.1 software (Analytical Software, Tallahassee, FL, USA). Mean variances of the data were analyzed using the least significant difference (LSD) test at *p* < 0.05. Graphs were plotted using Sigmaplot 10.0.

## 5. Conclusions

The present study revealed that Mo fertilizer plays a key role in N metabolism through regulating the activities and expressions of N-assimilating enzymes. Mo application decreased the NO_3_^−^ contents while increasing NO_2_^−^, NH_4_^+^, amino acids, and proteins contents in the leaf tissues, which is consistent with the enhanced NR, NiR, GS, and GOGAT activities and expression under different N sources; however, Mo-induced effects were highest under NH_4_NO_3_ source in both the wheat cultivars, suggesting that Mo plays a significant role in N assimilatory pathway and is more harmonious under mixture supply as NH_4_NO_3_ than either of the individual sources. Moreover, different N sources significantly interrupted the macro- and micro-elemental uptake, while Mo-induced effects were generally not significant. Interestingly, Mo-induced effects in the N metabolism enzymes activities and expressions and assimilatory products were more prominent in the ‘97014’ cultivar compared with the ‘97003’ cultivar, indicating that Mo has key importance in the N metabolism of winter wheat.

## Figures and Tables

**Table 1:** Influence of Mo supply on NO_3_^−^, NO_2_^−^, and NH_4_^+^ contents in leaves of Mo-efficient ‘97003’ and Mo-inefficient ‘97014’ winter wheat cultivars under different N sources.

Treatments		97003			97014		
		NO_3_^−^ Content (µmol·g^−1^FW)	NO_2_^−^ Content (µmol·g^−1^FW)	NH_4_^+^ Content (µmol·g^−1^FW)	NO_3_^−^ Content (µmol·g^−1^FW)	NO_2_^−^ Content (µmol·g^−1^FW)	NH_4_^+^ Content (µmol·g^−1^FW)
**NO_3_^−^**	−Mo	35.56 ± 5.32 ^a^	2.22 ± 0.22 ^c^	7.45 ± 0.57 ^c^	38.98 ± 4.19 ^a^	1.80 ± 0.10 ^b^	6.35 ± 0.82 ^b^
	+Mo	28.84 ± 2.78 ^ab^	2.74 ± 0.32 ^bc^	8.90 ± 1.06 ^c^	28.17 ± 4.27 ^bc^	2.53 ± 0.36 ^b^	8.19 ± 0.79 ^b^
**NH_4_NO_3_**	−Mo	28.04 ± 3.74 ^ab^	3.30 ± 0.43 ^b^	10.79 ± 1.22 ^bc^	33.04 ± 3.25 ^ab^	2.59 ± 0.21 ^b^	9.00 ± 0.65 ^b^
	+Mo	21.63 ± 1.73 ^b^	4.52 ± 0.53 ^a^	13.68 ± 1.38 ^ab^	21.44 ± 2.15 ^c^	4.18 ± 0.56 ^a^	12.85 ± 1.44 ^a^
**NH_4_^+^**	−Mo	1.70 ± 0.17 ^c^	0.34 ± 0.02 ^d^	13.43 ± 1.07 ^ab^	1.80 ± 0.22 ^d^	0.23 ± 0.04 ^c^	12.94 ± 1.32 ^a^
	+Mo	1.50 ± 0.08 ^c^	0.38 ± 0.04 ^d^	15.53 ± 1.94 ^a^	1.48 ± 0.12 ^d^	0.32 ± 0.04 ^c^	15.54 ± 1.80 ^a^

**Note:** −Mo and +Mo represent winter wheat cultivars fertilized, respectively, with 0 and 1 µM Mo [Na_2_MoO_4_.2H_2_O] against different N sources. Data represent means ± S.E from different independent treatments. Dissimilar superscripted letters (a, b, c, etc.) in each column indicate significant differences among different treatments at (*p* < 0.05). FW, fresh weight

**Table 2:** Influence of Molybdenum (Mo) supply on potassium, magnesium, and calcium concentrations in leaves of Mo-efficient ‘97003’ and Mo-inefficient ‘97014’ winter wheat cultivars under different N sources.

Treatments		97003			97014		
		K (mg·g^−1^DW)	Mg (mg·g^−1^DW)	Ca (mg g^−1^DW)	K (mg·g^−1^DW)	Mg (mg·g^−1^DW)	Ca (mg·g^−1^DW)
**NO_3_^−^**	**−Mo**	37.80 ± 1.62 ^a^	2.63 ± 0.19 ^ab^	12.09 ± 1.15 ^a^	37.31 ± 1.86 ^a^	2.14 ± 0.19 ^ab^	10.43 ± 1.49 ^ab^
	**+Mo**	34.30 ± 0.89 ^b^	3.25 ± 0.55 ^a^	13.79 ± 1.01 ^a^	35.49 ± 1.82 ^a^	2.80 ± 0.23 ^a^	12.92 ± 1.36 ^a^
**NH_4_NO_3_**	**−Mo**	30.89 ± 0.84 ^c^	2.24 ± 0.12 ^bc^	10.49 ± 0.76 ^a^	30.56 ± 0.64 ^b^	1.89 ± 0.20 ^b^	8.08 ± 0.86 ^bc^
	**+Mo**	28.24 ± 1.24 ^c^	2.87 ± 0.30 ^ab^	12.05 ± 1.63 ^a^	26.45 ± 1.39 ^c^	2.67 ± 0.43 ^a^	11.37 ± 0.78 ^a^
**NH_4_^+^**	**−Mo**	25.10 ± 0.55 ^d^	1.32 ± 0.15 ^d^	4.70 ± 0.23 ^b^	25.92 ± 0.63 ^c^	0.88 ± 0.04 ^c^	4.17 ± 0.29 ^d^
	**+Mo**	22.73 ± 0.74 ^d^	1.49 ± 0.15 ^cd^	5.27 ± 0.78 ^b^	23.71 ± 0.62 ^c^	1.09 ± 0.14 ^c^	4.85 ± 0.66 ^cd^

**Note:** −Mo and +Mo represent winter wheat cultivars fertilized, respectively, with 0 and 1 µM Mo [Na_2_MoO_4_.2H_2_O] against different N sources. Data represent means ± S.E from different independent treatments. Different superscripted letters (a, b, c, etc.) in each column indicate significant differences among different treatments at (*p* < 0.05). K, potassium; Mg, magnesium; Ca, calcium; and DW, dry weight.

**Table 3:** Influence of Molybdenum (Mo) supply on manganese, zinc, copper, and iron concentrations in leaves of Mo-efficient ‘97003’ and Mo-inefficient ‘97014’ winter wheat cultivars under different N sources.

Treatments		97003				97014			
		Mn (µg·g^−1^DW)	Zn (µg·g^−1^DW)	Cu (µg·g^−1^DW)	Fe (µg g^−1^DW)	Mn (µg·g^−1^DW)	Zn (µg·g^−1^DW)	Cu (µg·g^−1^DW)	Fe (µg·g^−1^DW)
**NO_3_^−^**	**−Mo**	72.78 ± 7.88 ^a^	26.51 ± 6.53 ^b^	7.83 ± 0.51 ^c^	84.19 ± 3.45 ^de^	69.28 ± 8.33 ^a^	30.34 ± 1.37 ^cd^	6.95 ± 0.53 ^c^	102.86 ± 22.01 ^bcd^
	**+Mo**	62.88 ± 7.34 ^ab^	22.39 ± 2.88 ^b^	8.44 ± 1.25 ^c^	71.04 ± 9.50 ^e^	55.53 ± 3.78 ^ab^	20.97 ± 2.88 ^d^	7.96 ± 1.09 ^c^	76.83 ± 6.28 ^d^
**NH_4_NO_3_**	**−Mo**	62.38 ± 10.05 ^ab^	32.23 ± 3.03 ^b^	9.89 ± 1.15 ^bc^	110.07 ± 20.12 ^bc^	60.64 ± 4.91 ^b^	34.18 ± 6.08 ^bc^	8.64 ± 0.84 ^c^	127.69 ± 15.12 ^bc^
	**+Mo**	50.93 ± 5.45 ^bc^	26.53 ± 4.33 ^b^	11.29 ± 1.57 ^abc^	96.82 ± 9.24 ^cd^	51.48 ± 7.54 ^bc^	23.93 ± 1.89 ^cd^	10.35 ± 1.69 ^bc^	99.18 ± 10.25 ^cd^
**NH_4_^+^**	**−Mo**	39.58 ± 3.60 ^cd^	52.35 ± 5.77 ^a^	15.14 ± 3.24 ^ab^	150.32 ± 11.47 ^a^	40.07 ± 4.31 ^cd^	54.63 ± 7.35 ^a^	14.58 ± 2.67 ^ab^	177.30 ± 21.16 ^a^
	**+Mo**	31.10 ± 4.50 ^d^	44.50 ± 5.78 ^a^	15.67 ± 1.69 ^a^	131.83 ± 15.88 ^ab^	31.06 ± 5.04 ^d^	41.77 ± 6.30 ^b^	15.71 ± 1.87 ^a^	141.69 ± 13.79 ^ab^

**Note:** −Mo and +Mo represent winter wheat cultivars fertilized, respectively, with 0 and 1 µM Mo [Na_2_MoO_4_.2H_2_O] against different N sources. Data represent means ± S.E from different independent treatments. Dissimilar superscripted letters (a, b, c, etc.) in each column indicate significant difference among different treatments at (*p* < 0.05). Mn, manganese; Zn, zinc; Cu, copper; and Fe, iron.

**Table 4:** The concentrations of salts (mM) used to prepare Hoagland nutrient solutions with dissimilar N sources NO_3_^−^, NH_4_:NO_3_, and NH_4_^+^.

Salts	NO_3_^−^	NH4:NO3	NH_4_^+^
Ca(NO_3_)_2_.4H_2_O	5.00	3.75	0.00
KNO_3_	5.00	0.00	0.00
CaCl_2_	0.00	1.25	5.00
K_2_SO_4_	0.00	2.50	2.50
(NH_4_)_2_SO_4_	0.00	3.75	7.50
MgSO_4_.7H_2_O	2.00	2.00	2.00
KH_2_PO_4_	1.00	1.00	1.00

Plastic pots (30 cm × 20 cm × 15 cm), containing four liters of respective solution and a perforated floating board on the surface of Hoagland solution with two rows for each cultivar, were used and each treatment was replicated four times. The pH was maintained at 6.5 ± 0.05 by the addition of HCl or NaOH to the nutrient solutions every day. The experimental pots were placed according to completely randomized design (CRD) with factorial arrangement.

**Table 5:** Sequences of primers used for qRT-PCR of *NR, NiR, GS,* and *GOGAT* genes.

Genes	Strand	Primer Sequence 5′ to 3′	Annealing Temperature (°C)
*NR*	Forward	CACCGGCCGCGGCAACTTC	58
	Reverse	CGAGACGGAGATGCACCTGG	
*NiR*	Forward	CGAGAAGAGGATGCCGAACG	56
	Reverse	CACGCCGAGGTAGTCACG	
*GS*	Forward	CACACCTCATCTCATCTCATCTC	52
	Reverse	TTCACCGTCCTTGCTTTGC	
*GOGAT*	Forward	TCATCCAGCCGACCAACACG	55
	Reverse	CCACAATCCATACAACGAGCAGAC	
*TaActin*	Forward	ACTGGGATGACATGGGGAA	55
	Reverse	ACCGCTGGCATACAAGGAC	

## References

[1] Castaings L., Marchive C., Meyer C., Krapp A. (2011). Nitrogen signalling in arabidopsis: How to obtain insights into a complex signalling network. J. Exp. Bot..

[2] Lam H.M., Coschigano K., Schultz C., Melooliveira R., Tjaden G., Oliveira I., Ngai N., Hsieh M.H., Coruzzi G. (1995). Use of arabidopsis mutants and genes to study amide amino acid biosynthesis. Plant Cell.

[3] Garg S. (2013). Role and hormonal regulation of nitrate reductase activity in higher plants: A review. Plant Sci. Feed.

[4] Nemie-Feyissa D., Królicka A., Førland N., Hansen M., Heidari B., Lillo C. (2013). Post-translational control of nitrate reductase activity responding to light and photosynthesis evolved already in the early vascular plants. J. Plant Physiol..

[5] Schwarz G., Mendel R.R., Ribbe M.W. (2009). Molybdenum cofactors, enzymes and pathways. Nature.

[6] Aslam M., Travis R.L., Rains D.W. (1996). Evidence for substrate induction of a nitrate efflux system in barley roots. Plant Physiol..

[7] Vanoni M., Curti B. (1999). Glutamate synthase: A complex iron-sulfur flavoprotein. Cell. Mol. Life Sci. Cmls.

[8] Nagy Z., Németh E., Guóth A., Bona L., Wodala B., Pécsváradi A. (2013). Metabolic indicators of drought stress tolerance in wheat: Glutamine synthetase isoenzymes and rubisco. Plant Physiol. Biochem..

[9] Pinto E., Fidalgo F., Teixeira J., Aguiar A.A., Ferreira I.M. (2014). Influence of the temporal and spatial variation of nitrate reductase, glutamine synthetase and soil composition in the n species content in lettuce (lactuca sativa). Plant Sci..

[10] Wang Z.-Q., Yuan Y.-Z., Ou J.-Q., Lin Q.-H., Zhang C.-F. (2007). Glutamine synthetase and glutamate dehydrogenase contribute differentially to proline accumulation in leaves of wheat (triticum aestivum) seedlings exposed to different salinity. J. Plant Physiol..

[11] Pajuelo P., Pajuelo E., Forde B.G., Márquez A.J. (1997). Regulation of the expression of ferredoxin-glutamate synthase in barley. Planta.

[12] Turano F.J., Muhitch M.J. (1999). Differential accumulation of ferredoxin-and nadh-dependent glutamate synthase activities, peptides, and transcripts in developing soybean seedlings in response to light, nitrogen, and nodulation. Physiol. Plant..

[13] Forde B.G. (2002). Local and long-range signaling pathways regulating plant responses to nitrate. Annu. Rev. Plant Biol..

[14] Chen M., Li K., Li H., Song C.P., Miao Y. (2017). The glutathione peroxidase gene family ingossypium hirsutum: Genome-wide identification, classification, gene expression and functional analysis. Sci. Rep..

[15] Min Y.U., Cheng-Xiao H.U., Sun X.C., Wang Y.H. (2010). Influences of mo on nitrate reductase, glutamine synthetase and nitrogen accumulation and utilization in mo-efficient and mo-inefficient winter wheat cultivars. Agric. Sci. China.

[16] Liu L., Xiao W., Li L., Li D.-M., Gao D.-S., Zhu C.-y., Fu X.-L. (2017). Effect of exogenously applied molybdenum on its absorption and nitrate metabolism in strawberry seedlings. Plant Physiol. Biochem..

[17] Mendel R.R., Schwarz G. (2011). Molybdenum cofactor biosynthesis in plants and humans. Coord. Chem. Rev..

[18] Kaiser B.N., Gridley K.L., Ngaire Brady J., Phillips T., Tyerman S.D. (2005). The role of molybdenum in agricultural plant production. Ann. Bot..

[19] Baxter I. (2009). Ionomics: Studying the social network of mineral nutrients. Curr. Opin. Plant Biol..

[20] Murgia I., Vigani G. (2015). Analysis of arabidopsis thaliana atfer4-1, atfh and atfer4-1/atfh mutants uncovers frataxin and ferritin contributions to leaf ionome homeostasis. Plant Physiol. Biochem..

[21] Baxter I.R., Vitek O., Lahner B., Muthukumar B., Borghi M., Morrissey J., Guerinot M.L., Salt D.E. (2008). The leaf ionome as a multivariable system to detect a plant’s physiological status. Proc. Natl. Acad. Sci..

[22] Na L., Li Z., Xiangxiang M., Ara N., Jinghua Y., Mingfang Z. (2014). Effect of nitrate/ammonium ratios on growth, root morphology and nutrient elements uptake of watermelon (citrullus lanatus) seedlings. J. Plant Nutr..

[23] Serna M., Borras R., Legaz F., Primo-Millo E. (1992). The influence of nitrogen concentration and ammonium/nitrate ratio on n-uptake, mineral composition and yield of citrus. Plant Soil.

[24] Roosta H.R., Schjoerring J.K. (2007). Effects of ammonium toxicity on nitrogen metabolism and elemental profile of cucumber plants. J. Plant Nutr..

[25] Sanchezechaniz J., Benitofernández J., Minteguiraso S. (2001). Methemoglobinemia and consumption of vegetables in infants. Pediatrics.

[26] Wright R.O., Lewander W.J., Woolf A.D. (1999). Methemoglobinemia: Etiology, pharmacology, and clinical management. Ann. Emerg. Med..

[27] Wen X., Hu C., Sun X., Zhao X., Tan Q. (2019). Research on the nitrogen transformation in rhizosphere of winter wheat (triticum aestivum) under molybdenum addition. Environ. Sci. Pollut. Res..

[28] Liu X., Zhao X., Li X., Chen S. (2019). Suppression of hesa mutation on nitrogenase activity in paenibacillus polymyxa wly78 with the addition of high levels of molybdate or cystine. PeerJ.

[29] Afridi M.M.R.K., Hewitt E.J. (1964). The inducible formation and stability of nitrate reductase in higher plants: I. Effects of nitrate and molybdenum on enzyme activity in cauliflower (brassica oleracea var. Botrytis). J. Exp. Bot..

[30] Spencer D., Wood J.G. (1954). The role of molybdenum in nitrate reduction in higher plants. Aust. J. Biol. Sci..

[31] Kovács B., Puskás-Preszner A., Huzsvai L., Lévai L., Bódi É. (2015). Effect of molybdenum treatment on molybdenum concentration and nitrate reduction in maize seedlings. Plant Physiol. Biochem..

[32] Balotf S., Kavoosi G., Kholdebarin B. (2016). Nitrate reductase, nitrite reductase, glutamine synthetase, and glutamate synthase expression and activity in response to different nitrogen sources in nitrogen-starved wheat seedlings. Biotechnol. Appl. Biochem..

[33] Balotf S., Islam S., Kavoosi G., Kholdebarin B., Juhasz A., Ma W. (2018). How exogenous nitric oxide regulates nitrogen assimilation in wheat seedlings under different nitrogen sources and levels. PLoS ONE.

[34] Xiao T., Mi M., Wang C., Meng Q., Chen Y., Zheng L., Zhang H., Hu Z., Shen Z., Yan X. (2018). A methionine-r-sulfoxide reductase, osmsrb5, is required for rice defense against copper toxicity. Environ. Exp. Bot..

[35] Keys A.J., Bird I.F., Cornelius M.J., Lea P.J., Wallsgrove R.M., Miflin B.J. (1978). Photorespiratory nitrogen cycle. Nature.

[36] Pérez-Delgado C.M., García-Calderón M., Márquez A.J., Betti M. (2016). Correction: Reassimilation of photorespiratory ammonium in lotus japonicus plants deficient in plastidic glutamine synthetase. PLoS ONE.

[37] Valadier M.H., Yoshida A., Grandjean O., Morin H., Kronenberger J., Boutet S., Raballand A., Hase T., Yoneyama T., Suzuki A. (2008). Implication of the glutamine synthetase/glutamate synthase pathway in conditioning the amino acid metabolism in bundle sheath and mesophyll cells of maize leaves. Febs J..

[38] Santi S., Locci G., Pinton R., Cesco S., Varanini Z. (1995). Plasma membrane h+-atpase in maize roots induced for no3-uptake. Plant Physiol..

[39] Campbell W. (2001). Structure and function of eukaryotic nad (p) h: Nitrate reductase. Cell. Mol. Life Sci. Cmls.

[40] Yu M., Hu C.-X., Wang Y.-H. (2002). Molybdenum efficiency in winter wheat cultivars as related to molybdenum uptake and distribution. Plant Soil.

[41] Yu M., Hu C., Wang Y. (1999). Influences of seed molybdenum and molybdenum application on nitrate reductase activity, shoot dry matter, and grain yields of winter wheat cultivars. J. Plant Nutr..

[42] Hachiya T., Watanabe C.K., Fujimoto M., Ishikawa T., Takahara K., Kawai-Yamada M., Uchimiya H., Uesono Y., Terashima I., Noguchi K. (2012). Nitrate addition alleviates ammonium toxicity without lessening ammonium accumulation, organic acid depletion and inorganic cation depletion in arabidopsis thaliana shoots. Plant Cell Physiol..

[43] Morozkina E., Zvyagilskaya R. (2007). Nitrate reductases: Structure, functions, and effect of stress factors. Biochem. (Mosc.).

[44] Peng L., Yu Y. (1999). Effect of molybdenum and boron on nitrogen metabolism of soybea. Plant Natrition Fertil..

[45] Calonego J.C., Junior E.U.R., Barbosa R.D., Leite G.H.P., Grassi Filho H. (2010). Adubação nitrogenada em cobertura no feijoeiro com suplementação de molibdênio via foliar. Rev. Ciência Agronômica.

[46] Goodall A.J., Kumar P., Tobin A.K. (2013). Identification and expression analyses of cytosolic glutamine synthetase genes in barley (hordeum vulgare l.). Plant Cell Physiol..

[47] Kronzucker H.J., Britto D.T., Davenport R.J., Tester M. (2001). Ammonium toxicity and the real cost of transport. Trends Plant Sci..

[48] Shelden M.C., Dong B., Guy L., Trevaskis B., Whelan J., Ryan P.R., Howitt S.M., Udvardi M.K. (2001). Arabidopsis ammonium transporters, atamt1; 1 and atamt1; 2, have different biochemical properties and functional roles. Plant Soil.

[49] Hachiya T., Sugiura D., Kojima M., Sato S., Yanagisawa S., Sakakibara H., Terashima I., Noguchi K. (2014). High co2 triggers preferential root growth of arabidopsis thaliana via two distinct systems under low ph and low n stresses. Plant Cell Physiol..

[50] Lambeck I.C., Fischer-Schrader K., Niks D., Roeper J., Chi J.-C., Hille R., Schwarz G. (2012). Molecular mechanism of 14-3-3 protein-mediated inhibition of plant nitrate reductase. J. Biol. Chem..

[51] Lea U.S., Leydecker M.-T., Quilleré I., Meyer C., Lillo C. (2006). Posttranslational regulation of nitrate reductase strongly affects the levels of free amino acids and nitrate, whereas transcriptional regulation has only minor influence. Plant Physiol..

[52] Miflin B.J., Habash D.Z. (2002). The role of glutamine synthetase and glutamate dehydrogenase in nitrogen assimilation and possibilities for improvement in the nitrogen utilization of crops. J. Exp. Bot..

[53] Wu S., Hu C., Tan Q., Nie Z., Sun X. (2014). Effects of molybdenum on water utilization, antioxidative defense system and osmotic-adjustment ability in winter wheat (triticum aestivum) under drought stress. Plant Physiol. Biochem..

[54] Hu C., Wang Y., Wei W. (2002). Effect of molybdenum applications on concentrations of free amino acids in winter wheat at different growth stages. J. Plant Nutr..

[55] Engels C., Marschner H. (1993). Influence of the form of nitrogen supply on root uptake and translocation of cations in the xylem exudate of maize (zea mays l). J. Exp. Bot..

[56] Helali S.M.r., Nebli H., Kaddour R., Mahmoudi H., Lachaâl M., Ouerghi Z. (2010). Influence of nitrate—Ammonium ratio on growth and nutrition of arabidopsis thaliana. Plant Soil.

[57] Van Beusichem M.L., Kirkby E.A., Baas R. (1988). Influence of nitrate and ammonium nutrition on the uptake, assimilation, and distribution of nutrients in ricinus communis. Plant Physiol..

[58] Vigani G., Di Silvestre D., Agresta A.M., Donnini S., Mauri P., Gehl C., Bittner F., Murgia I. (2017). Molybdenum and iron mutually impact their homeostasis in cucumber (cucumis sativus) plants. New Phytol..

[59] Nie Z., Hu C., Liu H., Tan Q., Sun X. (2014). Differential expression of molybdenum transport and assimilation genes between two winter wheat cultivars (triticum aestivum). Plant Physiol. Biochem..

[60] Sun X., Hu C., Tan Q., Liu J., Liu H. (2009). Effects of molybdenum on expression of cold-responsive genes in abscisic acid (aba)-dependent and aba-independent pathways in winter wheat under low-temperature stress. Ann. Bot..

[61] Wu S., Hu C., Tan Q., Xu S., Sun X. (2017). Nitric oxide mediates molybdenum-induced antioxidant defense in wheat under drought stress. Front. Plant Sci..

[62] Rao L.V.M., Rajasekhar V.K., Sopory S.K., Guhamukherjee S. (1981). Phytochrome regulation of nitrite reductase—A chloroplast enzyme—In etiolated maize leaves. Plant Cell Physiol..

[63] Rhodes D., Rendon G.A., Stewart G.R. (1975). The control of glutamine synthetase level in lemna minor L.. Planta.

[64] Lin C.C., Kao C.H. (1996). Disturbed ammonium assimilation is associated with growth inhibition of roots in rice seedlings caused by nacl. Plant Growth Regul..

[65] Pfaffl M.W. (2001). A new mathematical model for relative quantification in real-time rt–pcr. Nucleic Acids Res..

[66] Cataldo D., Maroon M., Schrader L., Youngs V. (1975). Rapid colorimetric determination of nitrate in plant tissue by nitration of salicylic acid 1. Commun. Soil Sci. Plant Anal..

[67] Molins-Legua C., Meseguer-Lloret S., Moliner-Martinez Y., Campíns-Falcó P. (2006). A guide for selecting the most appropriate method for ammonium determination in water analysis. Trac Trends Anal. Chem..

[68] Hamilton P.B., Van Slyke D.D., Lemish S. (1943). The gasometric determination of free amino acids in blood filtrates by the ninhydrin-carbon dioxide method. J. Biol. Chem..

[69] Bradford M.M. (1976). A rapid method for the quantitation of microgram quantities of protein utilizing the principle of protein-dye binding. Anal. Biochem..

